# Coronary Artery Calcium - From Screening to a Personalized Shared Decision-Making Tool: The New American Prevention Guidelines

**DOI:** 10.5935/abc.20190008

**Published:** 2019-01

**Authors:** Marcio Sommer Bittencourt, Michael J. Blaha, Khurram Nasir

**Affiliations:** 1 Centro de Pesquisa Clínica e Epidemiológica do Hospital das Clínicas da Universidade de São Paulo, São Paulo, SP - Brazil; 2 Hospital Israelita Albert Einstein e Faculdade Israelita de Ciências da Saúde Albert Einstein, São Paulo, SP - Brazil; 3 The Johns Hopkins Ciccarone Center for the Prevention of Heart Disease, Baltimore, Maryland - USA; 4 Center for Outcomes Research and Evaluation, Yale-New Haven Hospital, New Haven, Connecticut – USA

**Keywords:** Cardiovascular Diseases/prevention and control, Dyslipidemias/prevention and control, Health Care/Public Health), Decision Making, Cholesterol,LDL, Risk Factors, Calcium Score

The United States (US) National Cholesterol Education Program (NCEP) formed the Adult Treatment Panel (ATP) in 1985 with the aim to educate clinician and provide guideline recommendations for the treatment of dyslipidemias. In its first 1998 recommendations, the approach to primary prevention of cardiovascular disease included LDL-cholesterol (LDL-C) reduction in individuals with more than two risk factors and LDL-C levels above 160mg/dL and optional treatment in those with “borderline” LDL-C levels between 130 - 159 mg/dL.^1^ In its second version, in 1994, a category of secondary prevention with a target LDL-C below 100mg/dL was introduced.^2^ In 2001 the third version of the document, ATP-III introduced the concept of “optimal” LDL-C < 100mg/dL and introduced the use of the 10-year Framingham risk score (FRS) for the estimation of risk to define the intensity of treatment and target LDL-C levels,^3^ and an update of this document introduced a more aggressive LDL-C < 70 mg/dL target for those at extremely high risk. The ATP-III also mentions coronary artery calcium (CAC) as an “emerging risk factor”, stating it could be of value for additional risk stratification, predominantly in intermediate risk groups. Interestingly, at this point the recommendations were that CAC could be of use in individuals with multiple risk factors or older individuals in whom “traditional risk factors lose some of their predictive power”. In both cases CAC was proposed as a tool to screen for individuals at an even higher than expected risk, though the ATP-III clearly advised against the widespread use of CAC as a screening tool.

After the update of ATP-III there was a considerable gap before the publication of the 2013 ACC/AHA Blood Cholesterol guidelines,^4^ and a completely new approach towards the selection of candidates for treatment of LDL-C was taken. First, this document updated the equations for calculating 10-year cardiovascular risk (the Framingham Risk Score only predicted risk of coronary heart disease). Second, it identified higher risk groups which should be treated irrespective of risk (LDL-C > 190 mg/dL, diabetics). Third, it proposed a much broader recommendation of statin use for primary prevention including all individuals with LDL-C > 70 mg/dL and a calculated atherosclerotic cardiovascular disease risk > 5% in 10 years. This broader approach has been criticized by many , as it resulted in a substantial increase in the number of individuals in whom statins would be recommended,^5^ including treatment recommendations of lower risk individuals due to overestimation of risk derived from the risk assessment tool.^6^ This document also gave CAC a class IIb indication for a selective use in individuals fitting the vague description “in whom the decision to start treatment was unclear”. In this document additional markers of risk included CAC, high sensitivity C-reactive protein, ankle braquial index, family history of premature cardiovascular disease or individuals with LDL-C > 160 mg/dL - and all were given similar IIb recommendations with little to differentiate their predictive power. As recommended in ATP-III, the use of those markers was as an additional screening tool to identify individuals with a higher risk for more aggressive treatment, though no clear recommendation on its use was provided.

The history of treatment recommendation of dyslipidemias in primary prevention in Brazil follows a similar pattern, with an initial consensus published in 1994 where basic definitions of dyslipidemias were given but no clear recommendations or LDL-C targets were defined. In its fifth recommendation, the Brazilian Society of Cardiology included for the first time the use of CAC as an “aggravating” risk factor, and suggested a more aggressive treatment of individuals with CAC > 100 or above the 75^th^ percentile,^7^ yet this was still a recommendation of CAC as a screening tool to identify higher risk individuals.

The most recent update of the US recommendations provided several changes to prior recommendations. First, an intermediate risk group was reincorporated as part of the risk stratification. In the new guidelines individuals with a 10 year risk < 5% are considered low risk, those between 5-7.5% are considered borderline, those between 7.5-20% are considered intermediate risk and those above 20% are considered high risk individuals. The recognition of borderline and intermediate risk groups can be interpreted as a need to recognize the considerable uncertainty let from the risk estimations currently used in practice. While treatment strategies are probably well defined for the majority of individuals in the extremes of risk, a considerable proportion of the population still lies in the two “gray zone” groups were uncertainty in the recommendation may arise during the clinician-patient risk discussion.

This, in fact, highlights another aspect of the new guidelines. The document highlights the need for shared decision making before any new medication prescription including a discussion of risks and benefits of pharmacological and non-pharmacological treatment strategies. Particularly for those individuals at intermediate, and maybe borderline, risk one may expect considerable uncertainty in the need for therapy for many individuals. For this group of patients, the guidelines recommend considering additional risk factors as potential tools to favor pharmacological treatment.

For the use of CAC, a completely new approach has been proposed. Instead of a tool used only to selected higher risk individuals in whom treatment should be more aggressive, CAC is now proposed as a two-way tool (can move individuals both up and down the risk spectrum) for individuals in who treatment might be considered. On the one hand, if CAC = 0, pharmacologic treatment can be withheld or delayed for most individuals, whereas CAC > 0 favors treatment, particularly if > 100 units, > 75^th^ percentile or if > 0 in individuals younger than 55 years old.

This unique ability of CAC to “derisk” individuals of intermediate risk is not trivial. In this group approximately, half of the population has a CAC = 0 and could be withheld for treatment for a considerable follow up.^8^ Based on these new recommendations, a considerable reduction in the need for treatment can be anticipated in CAC is implemented as recommended. Interestingly, some data suggests that this approach can be cost effective from a societal perspective.^9^

Still, some gaps in knowledge still remain for the widespread use of this strategy. First, the guidelines highlight that this approach might not be recommended in diabetics, smokers and individuals with a history of premature cardiovascular disease, though this is largely based on the limited data available for those subgroups rather than on evidence of harm. Second, this approach is not supported by randomized clinical trial, though trials in this area have been proposed. While some have also cautioned on the use of radiation, the current exposure from a CAC scan (0.89 mSv), less than one third of the annual background radiation exposure. Finally, a major gap in the widespread use of CAC both in the US and in Brazil is the current lack of reimbursement by most health care providers or the public system in Brazil.

Despite those areas in need of further study and challenges in implementation, the new approach towards individualized risk assessment and shared decision making with the optional inclusion of CAC as part of the decision-making toolkit is a huge step toward a more precise treatment targeted at the individual’s preferences.
